# Is Cancer Cachexia Attributed to Impairments in Basal or Postprandial Muscle Protein Metabolism?

**DOI:** 10.3390/nu8080499

**Published:** 2016-08-16

**Authors:** Astrid M. H. Horstman, Steven W. Olde Damink, Annemie M. W. J. Schols, Luc J. C. van Loon

**Affiliations:** 1 Departments of Human Biology and Movement Sciences, NUTRIM School of Nutrition and Translational Research in Metabolism, Maastricht University, P.O. Box 616, Maastricht 6200 MD, The Netherlands; astrid.horstman@maastrichtuniversity.nl; 2 Departments of Surgery, NUTRIM School of Nutrition and Translational Research in Metabolism, Maastricht University, P.O. Box 616, Maastricht 6200 MD, The Netherlands; steven.oldedamink@maastrichtuniversity.nl; 3 Respiratory Medicine, NUTRIM School of Nutrition and Translational Research in Metabolism, Maastricht University, P.O. Box 616, Maastricht 6200 MD, The Netherlands; a.schols@maastrichtuniversity.nl

**Keywords:** muscle metabolism, lean body mass, nutrition, protein, FSR, FBR, postprandial, anabolic resistance

## Abstract

Cachexia is a significant clinical problem associated with very poor quality of life, reduced treatment tolerance and outcomes, and a high mortality rate. Mechanistically, any sizeable loss of skeletal muscle mass must be underpinned by a structural imbalance between muscle protein synthesis and breakdown rates. Recent data indicate that the loss of muscle mass with aging is, at least partly, attributed to a blunted muscle protein synthetic response to protein feeding. Whether such anabolic resistance is also evident in conditions where cachexia is present remains to be addressed. Only few data are available on muscle protein synthesis and breakdown rates in vivo in cachectic cancer patients. When calculating the theoretical changes in basal or postprandial fractional muscle protein synthesis and breakdown rates that would be required to lose 5% of body weight within a six-month period, we can define the changes that would need to occur to explain the muscle mass loss observed in cachectic patients. If changes in both post-absorptive and postprandial muscle protein synthesis and breakdown rates contribute to the loss of muscle mass, it would take alterations as small as 1%–2% to induce a more than 5% decline in body weight. Therefore, when trying to define impairments in basal and/or postprandial muscle protein synthesis or breakdown rates using contemporary stable isotope methodology in cancer cachexia, we need to select large homogenous groups of cancer patients (>40 patients) to allow us to measure physiological and clinically relevant differences in muscle protein synthesis and/or breakdown rates. Insight into impairments in basal or postprandial muscle protein synthesis and breakdown rates in cancer cachexia is needed to design more targeted nutritional, pharmaceutical and/or physical activity interventions to preserve skeletal muscle mass and, as such, to reduce the risk of complications, improve quality of life, and lower mortality rates during the various stages of the disease.

## 1. Introduction

The rapid weight loss that accompanies cancer is known as cancer cachexia. Cachexia is associated with impaired physical function [1], reduced tolerance to anticancer therapy [2], and increased mortality [3]. The loss of muscle mass can be profound, sometimes reaching as much as 75%, and is the most apparent feature of cancer cachexia [4,5]. Cachexia affects ~50% of all cancer patients, may account for ~20%–25% of all cancer deaths, and is one of the primary causes of asthenia, respiratory complications, poor response to chemotherapy, increased susceptibility to infection, and poor quality of life [6,7]. However, weight loss in patients with cancer is rarely recognized, assessed [8], or managed actively [9]. In short, cancer cachexia requires more attention and should be counteracted. 

Cancer cachexia was recently defined as “a multifactorial syndrome characterized by an ongoing loss of skeletal muscle mass (with or without loss of fat mass) that cannot be fully reversed by conventional nutritional support and leads to progressive functional impairment” [10]. The loss of adipose tissue often precedes lean body mass loss and may contribute to the multiple co-morbidities that have been associated with cancer cachexia [11,12]. Patients who have lost more than 5% of (stable) their body weight over the past six months, or have a body mass index (BMI) less than 20 kg/m^2^ and ongoing weight loss of more than 2%, or show an appendicular skeletal muscle index consistent with sarcopenia and any degree of weight loss >2% are generally classified as suffering from cachexia. This consensus definition [10] reflects the complex interplay between reduced food intake and metabolic impairments and identifies the loss of skeletal muscle mass as being key in the development of functional and metabolic impairments in patients. This supports the concept that skeletal muscle mass can be regarded as both a marker for the syndrome as well as an important therapeutic target. Hence, the consensus extends beyond the mere observation of weight loss and warrants direct measures of body composition and the assessment of skeletal muscle mass.

Mechanistically, any sizeable loss of skeletal muscle mass must be underpinned by a persistent imbalance between muscle protein synthesis and breakdown rates. In the cancer patient the balance between protein synthesis and breakdown may be disrupted by the tumor(s), the disease burden, and the various therapeutic interventions. Unfortunately, only few data are available on the impact of tumor burden and the stage of the disease and its treatment on basal or postprandial muscle protein synthesis and breakdown rates in vivo in cachectic cancer patients [13,14,15,16]. 

## 2. Post-Absorptive Muscle Protein Metabolism

Post-absorptive muscle protein synthesis and breakdown rates can be assessed in vivo in humans by the use of contemporary stable isotope methodology [17,18]. Based upon a 5% body weight loss within a six-month period, we can calculate the theoretical changes in basal muscle protein synthesis or breakdown rates that would be required to induce such a decline in muscle mass. Assuming that the 5% decline in body weight is fully attributed to the loss of lean mass, this implies that an average lean man (body weight: 75 kg; lean mass: 45 kg) would have lost ~4 kg lean tissue within a six-month period. This would represent a ~9% decline in muscle mass over a six-month period (182.5 days, 4380 h) or a rate of muscle loss of 0.002%/h. With basal muscle protein synthesis and breakdown rates ranging between 0.03% and 0.06%/h [17,19], such a rapid decline in muscle mass could already be induced by either a 3%–6% decrease in post-absorptive muscle protein synthesis or a similar-sized increase in post-absorptive muscle protein breakdown rates. Clearly, when muscle wasting is attributed to both a decline in basal muscle protein synthesis as well as a similar-sized increase in proteolysis, a mere 2%–3% change in both protein synthesis and breakdown rates would be sufficient to explain a 5% lean mass loss. Using contemporary stable isotope methodology, such small changes can only be assessed using an appropriate number of patients from a relatively homogenous cohort of patients experiencing the same type of cancer, stage of the disease, degree of inflammation and/or level of insulin resistance. If post-absorptive muscle protein synthesis rates would average 0.030% ± 0.003%/h and 0.028% ± 0.003%/h in such a patient cohort and healthy control group, respectively, a sample size of at least 37 subjects per group would be required to detect statistically significant differences between groups (with a power of 0.8 and a level of significance of 0.05). Therefore, to evaluate whether changes in post-absorptive muscle protein synthesis and/or breakdown rates contribute to the muscle wasting observed in cachectic cancer patients, relatively large (homogenous) cohorts of as much as ~40 patients and a similar amount of age-matched, healthy controls need to be recruited in the study. 

## 3. Postprandial Muscle Metabolism

Over the last decade it has become evident that differences in basal muscle protein synthesis and/or breakdown rates represent only part of the regulatory processes that define muscle mass preservation. Recent work has shown that loss of muscle mass with surgery [20], injury [21], disuse [22], aging [23,24] and disease [25] is, at least partly, attributed to a reduced sensitivity of skeletal muscle tissue to the anabolic properties of (protein) feeding. Muscle protein synthesis and breakdown rates are highly responsive to amino acid or protein ingestion [18], with a substantial increase in muscle protein synthesis rates [26] and accompanying decline in muscle protein breakdown [18] following feeding. Though cancer patients still show a muscle protein synthetic response to protein ingestion [13,14,15,16,27], there is ample evidence to show that in cancer cachexia, the responsiveness to protein administration is strongly reduced [13,16,27]. This anabolic resistance to feeding attenuates the postprandial rise in muscle protein synthesis and most likely contributes to the loss of muscle mass observed in cancer cachexia. To calculate the theoretical changes in postprandial muscle protein synthesis rates that would be required to lose 5% of body weight (or 9% of muscle mass) within a six-month period, we first assume a postprandial state for ~4 h following each of the three main daily meals, resulting in a total postprandial period of 12 h out of 24 h per day. For the muscle mass loss to occur within those 12 h per 24 h throughout a six-month period, the postprandial muscle protein synthetic response to feeding should be blunted by 0.004%/h. 

With a normal postprandial muscle protein synthesis rate ranging between 0.04 and 0.05%/h [19,28], a 0.004%/h decline in the postprandial muscle protein synthesis rate would represent an 8%–10% decline in the muscle protein synthetic response to feeding. If postprandial muscle protein synthesis rates would average 0.046% ± 0.006%/h and 0.050% ± 0.006%/h in a patient cohort and healthy control group, respectively, a sample size of at least 37 subjects per group would be required to detect statistically significant differences between groups (with a power of 0.8 and a level of significance of 0.05). Therefore, relatively large (homogenous) groups of patients and healthy controls will need to be recruited to detect statistically significant impairments in postprandial protein handling in cachectic cancer patients compared with healthy controls. 

Only a few studies have assessed fractional muscle protein synthesis [13,14,15,16] or breakdown [14] rates in cancer patients (see Table 1). Despite the relative lack of data, these studies suggest that changes in both post-absorptive and postprandial muscle protein synthesis rates may contribute substantially to muscle wasting in cachectic cancer patients. However, muscle loss with cancer can also be attributed to increases in muscle protein breakdown rates. It has been suggested that the primary factor driving cancer cachexia is enhanced protein degradation through the ubiquitin-proteasome pathway [7,29]. However, only one case study has tried to measure post-absorptive fractional muscle protein breakdown rates in cancer patients [14]. Although it has never been assessed, it is evident that both an increase in the basal muscle protein breakdown rate as well as a blunted postprandial decline in muscle protein breakdown may contribute considerably to the observed loss of lean mass in cancer cachexia [14]. Identical to the calculations for the postprandial increase in muscle protein synthesis, an 8%–10% attenuation of the postprandial decline in muscle protein breakdown would suffice to induce a 5% loss in body weight within a six-month period. In case the muscle loss is accompanied by both a decline in the postprandial rise in the muscle protein synthesis rate as well as an attenuated decline in muscle protein breakdown, changes in postprandial protein handling as small as 4%–5% could result in a 5% loss of body weight. As presented above, relatively large groups of patients and similar-sized healthy controls will need to be selected to allow us to detect statistically significant differences in postprandial protein synthesis and breakdown rates between groups. Such large studies will be required to provide clinical evidence of anabolic resistance as a key factor in muscle wasting in cancer cachexia.

## 4. Conclusions and Future Directions

**Figure 1:**
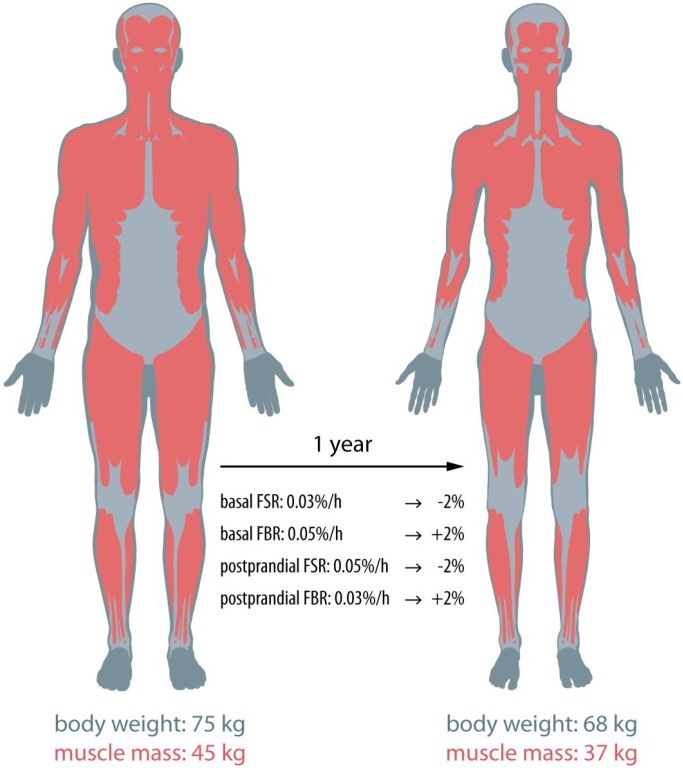
Small changes (1%–2%) in basal and postprandial fractional muscle protein synthesis (FSR) and breakdown (FBR) rates can result in substantial muscle mass loss within a year.

Despite a lack of data, it is likely that changes in both basal and postprandial muscle protein synthesis and breakdown rates contribute to the muscle wasting observed in cachectic cancer patients. As is evident from the theoretical evaluation above, if all of these factors contribute equally to the loss of lean mass, mere 1%–2% changes in post-absorptive and postprandial muscle protein synthesis and breakdown rates would suffice to induce substantial muscle loss (Figure 1). Such small differences in muscle protein synthesis or breakdown rates are far too small to be evaluated with contemporary stable isotope methodology in (heterogeneous) patient cohorts of 10–20 patients. However, it should be noted that in some cases, much greater losses in muscle mass are observed in shorter time frames, making observations of changes in basal or postprandial protein metabolism more evident [31,32,33,34]. 

To preserve muscle mass and function in cancer patients and, as such, counteract cancer cachexia, we need to know what regulatory processes contribute to the observed muscle loss. Muscle loss may be attributed to small but consistent changes in the post-absorptive muscle protein synthesis and breakdown rates as well as in the capacity of muscle protein synthesis and breakdown to respond adequately to anabolic stimuli, such as feeding and physical activity [35]. Insight into these impairments in the balance between basal and/or postprandial muscle protein synthesis and breakdown rates will allow us to design targeted treatments that can help to preserve skeletal muscle mass and function in cancer cachexia and, as such, reduce the risk of complications, improve quality of life and lower mortality rates during the different stages of the disease and its treatment.

## Figures and Tables

**Table 1:** Muscle protein synthesis and breakdown rates in cancer patients.

	Methods Used	Basal FSR (%/h)	Postprandial FSR (%/h)	Basal FBR (%/h)	Nutritional Intervention
Deutz, 2011 [13]	Primed continuous infusion l-[ring-^13^C_6_]-Phe	0.073 ± 0.023 ^a^ 0.073 ± 0.022 ^b^	0.097 ± 0.033 ^a^ 0.065 ± 0.028 ^b^	-	^a^ conventional medical food ^b^ re-designed medical food
Dillon, 2012 (case report) [14]	Pulse bolus injection [30] l-[ring-^13^C_6_]-Phe and ^15^N-Phe	0.07	-	0.03	-
Dillon, 2007 [15]	Primed continuous infusion l-[ring-^2^H_5_]-Phe	0.052 ± 0.009	0.120 ± 0.008	-	amino acid supplement
Williams, 2012 [16]	Primed continuous infusion [1,2-^13^C_2_]-Leu and ring-D5-Phe	0.028 ± 0.004	0.038 ± 0.004	-	intravenous mixed amino acids

Phe: phenylalanine; Leu: leucine; FSR: fractional synthetic rate; FBR: fractional breakdown rate; ^a^ Conventional medical food; ^b^ Re-designed medical food.
